# Designing a typhoid environmental surveillance study: A simulation model for optimum sampling site allocation

**DOI:** 10.1016/j.epidem.2020.100391

**Published:** 2020-06

**Authors:** Yuke Wang, Christine L. Moe, Shanta Dutta, Ashutosh Wadhwa, Suman Kanungo, Wolfgang Mairinger, Yichuan Zhao, Yi Jiang, Peter FM. Teunis

**Affiliations:** a Center for Global Safe Water, Sanitation, and Hygiene, Hubert Department of Global Health, Rollins School of Public Health, Emory University, Atlanta, GA, USA; b National Institute of Cholera and Enteric Diseases, Kolkata, India; c Department of Mathematics and Statistics, Georgia State University, Atlanta, GA, USA

**Keywords:** Disease surveillance, Environmental surveillance, Typhoid fever, Sampling strategy, Adaptive sampling, Mathematical modeling

## Abstract

Environmental surveillance can be used for monitoring enteric disease in a population by detecting pathogens, shed by infected people, in sewage. Detection of pathogens depends on many factors: infection rates and shedding in the population, pathogen fate in the sewerage network, and also sampling sites, sample size, and assay sensitivity. This complexity makes the design of sampling strategies challenging, which creates a need for mathematical modeling to guide decision making.

In the present study, a model was developed to simulate pathogen shedding, pathogen transport and fate in the sewerage network, sewage sampling, and detection of the pathogen. The simulation study used *Salmonella enterica* serovar Typhi (*S.* Typhi) as the target pathogen and two wards in Kolkata, India as the study area. Five different sampling strategies were evaluated for their sensitivity of detecting *S.* Typhi, by sampling unit: sewage pumping station, shared toilet, adjacent multiple shared toilets (primary sampling unit), pumping station + shared toilets, pumping station + primary sampling units. Sampling strategies were studied in eight scenarios with different geographic clustering of risk, pathogen loss (decay, leakage), and sensitivity of detection assays. A novel adaptive sampling site allocation method was designed, that updates the locations of sampling sites based on their performance. We then demonstrated how the simulation model can be used to predict the performance of environmental surveillance and how it is improved by optimizing the allocation of sampling sites.

The results are summarized as a decision tree to guide the sampling strategy based on disease incidence, geographic distribution of risk, pathogen loss, and the sensitivity of the detection assay. The adaptive sampling site allocation method consistently outperformed alternatives with fixed site locations in most scenarios. In some cases, the optimum allocation method increased the median sensitivity from 45% to 90% within 20 updates.

## Graphical abstract

**Figure:**
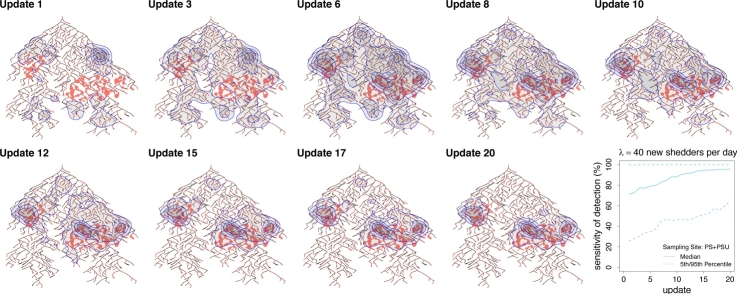

## 1 Introduction

The estimated global deaths caused by typhoid fever decreased from 600,000 in 1984 to between 128,200 and 161,000 in 2017. However, typhoid remains a major cause of morbidity especially in South Asia and sub-Saharan Africa (Crump et al., 2004, World Health Organization, 2018, Bhutta et al., 2018). Epidemics of *Salmonella enterica* serovar Typhi (*S.* Typhi) have been reported in Kolkata, India since 1990 (Saha et al., 1992), and typhoid fever is endemic in Kolkata with estimated incidence in 2004 of 1.6 cases per 1,000 population per year (Sur et al., 2006). Few low- and middle-income countries have a national surveillance system for typhoid fever. In Kolkata, active clinical surveillance for typhoid was conducted in Wards 29 and 30 (Municipal wards of Kolkata Wikipedia Page, 2018, Kolkata Municipal Corporation Website, 2018) in 2004 (Sur et al., 2006) and is ongoing in Wards 58 and 59 (Municipal wards of Kolkata Wikipedia Page, 2018, Kolkata Municipal Corporation Website, 2018) since Nov 2017 by the Indian Council of Medical Research-National Institute of Cholera and Enteric Diseases (ICMR-NICED). However, maintaining large-scale active clinical surveillance for typhoid fever requires tremendous resources.

Environmental surveillance (ES) is a strategy to monitor the circulation of specific enteric pathogens in a population by examining sewage samples containing human feces (Hovi et al., 2012). While clinical surveillance detects symptomatic cases, ES detects pathogen shedding, including silent transmission, which cannot be directly observed. ES plays a key role in the global polio eradication campaign and has been used to monitor the effectiveness of polio vaccination programs. As circulation of poliovirus dramatically decreased, clinical surveillance for acute flaccid paralysis has become inadequate because of the high proportion of asymptomatic infections, non-specific clinical presentation, and challenges with laboratory confirmation (Hovi et al., 2012). Several countries, including Egypt, Israel, Pakistan, India, and Nigeria, have deployed ES for polio, and detection of poliovirus circulation by ES in the absence of clinical cases (“silent outbreaks”) has been reported (Manor et al., 1999, Asghar et al., 2014). Such findings demonstrate how ES may play an important role in monitoring disease transmission and complements clinical surveillance.

In 2003, WHO published the “Guidelines for environmental surveillance of poliovirus circulation”, which recommends that sampling sites be located at inlets of sewage treatment plants or other major collector sewers (World Health Organization, 2003). Such a strategy works well when the sewerage system serves most of the target population (including the poor) and consistently carries human fecal material, the target pathogen has prolong environmental persistence, and it is easy to detect the target pathogen in sewage samples. However, in situations with no sewerage network, inconsistent sewage flow, rapid pathogen decay, substantial pathogen loss, or large dilution of pathogens in the sewerage system, combined with detection assays that are not very sensitive or specific, sampling at sites distant from shedding sources may fail to detect the target pathogen. Unlike poliovirus, *S.* Typhi is likely to be less persistent in the environment and more challenging to detect in environmental samples. When the infection pressure, geographic distribution of risk, and the structure and coverage of the sewerage network are not known, a model is critical to guide the sampling strategy, particularly sampling sites allocation, because of the risk that ES may fail to detect circulation of *S.* Typhi.

Ranta et al. (2001) explored the sensitivity of polio ES by means of a mathematical model, and compared sensitivities under different transmission scenarios (endemic vs. epidemic). The current study models the fate of *S.* Typhi in the sewerage network in two wards of Kolkata, India and compares the sensitivities of different sampling strategies for relevant transmission scenarios (e.g. small vs. large pathogen loss in the environment). The objectives of this study are: (1) develop a mathematical model to simulate fecal shedding dynamics and pathogen fate in the sewerage network; (2) compare sensitivities of different sewage sampling strategies in different scenarios; (3) develop an adaptive method for ES sampling site allocation to optimize the likelihood of detecting the target pathogen if it is circulating in the community.

## 2 Methods and simulations

### 2.1 Ward 58 and 59 of Kolkata, India

The current study is part of an effort to develop a typhoid ES strategy for Kolkata, India. For the simulation study, we selected settings and parameters based on information on Ward 58 (22.543333° N 88.39725° E) and Ward 59 (22.545333° N 88.377125° E) (Kolkata Municipal Corporation Website, 2018, Municipal wards of Kolkata Wikipedia Page, 2018, Cook et al., 2008) in Kolkata, India, where typhoid ES data collection is planned for 2019. Wards 58 and 59 have a population size of about 160,000 people in about 30,000 households (unpublished data). Most underground sewage passes through a pumping station (PS) located at the North boundary of Wards 58 and 59. Active typhoid clinical surveillance amongst about 6000 children in three age groups (6 month–4 years old, 5–9 years old, 10–14 years old) has been conducted by the National Institute of Cholera and Enteric Diseases (NICED) since November 2017.

### 2.2 Sewerage system

The sewerage system infrastructure conveys sewage and surface runoff (stormwater) out of the city. Generally, the system consists of receiving drains, a network of pipes, pumping stations, and access points (manholes) (Sewerage Wikipedia Page, 2018, UN Water, 2015). Sanitary sewers (UN Water, 2015) transport fecal matter from toilets in houses and commercial buildings to treatment facilities or disposal sites. The sewerage network can be either above ground or underground with separate sanitary sewers and storm drains or combined sewers that collects both sewage and surface runoff. Sewerage networks, either simplified, conventional, or condominial (Seyoum, 2016), usually have a tree topology with many branches that converge to the main trunk line (with the outdegree of the sewerage network close to 1 (Novel-T Innovative Solution, 2018)). Communities in Kolkata need to regularly remove solid trash blocking the sewer to avoid overflow during periods of increased rainfall. This suggests that there are no alternative flow paths and supports the assumption of a tree topology. Household toilets are the entry points (sources) in the network and a pumping station is the endpoint (sink).

**Fig. 1:**
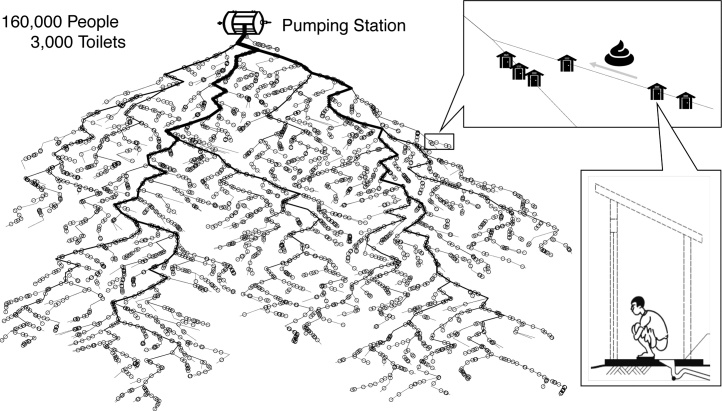
Diagram of feces flow from toilets to pumping station through the underground sewerage network, which is a hypothetical network created based on certain characteristics (topology, number of shared toilets, number of pumping station, size of catchment areas, etc.) of sewerage network in Ward 58 and 59 of Kolkata, India.

The sewerage system in Kolkata is a combined underground system, and corresponding settings were used for the simulation model. The exact structure of the sewerage network in Wards 58 and 59 is undocumented. Therefore, in this simulation study, the sewerage structure, including upstream and downstream relationship, was assumed unknown. Sewerage networks with 1000 edges (links between two nodes) were simulated using the SSN package (Hoef et al., 2014) in R (R Core Team, 2015). The water use per capita in Kolkata is 200 liters (L) per day with an average 35% water loss (Basu, 2015), which corresponds to approximately $2\times 10^{7}$ L water in total transported daily through the pumping station. The simulated sewerage network is a directed weighted network, and the edge weight represents the fraction of the total volume flow passing through the edge (Fig. 1). It was estimated that an average of 10 households (with an average size of 5 people) share a single toilet in Kolkata. Thus, 3000 shared toilets, as entry points of fecal pathogens into the sewerage system, were assigned based on a binomial process with equal probability for any point in the network.

### 2.3 Pathogen shedding kinetics

**Fig. 2:**
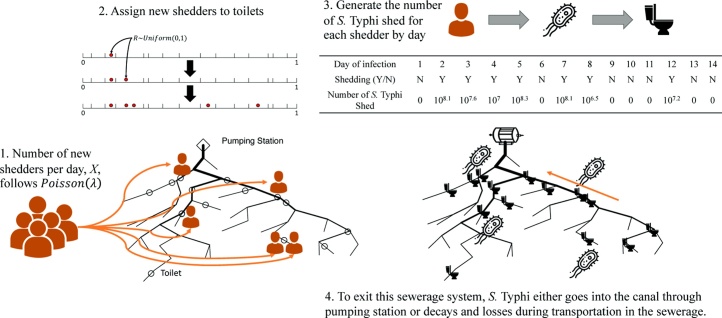
Life-cycle diagram of *S.* Typhi in the sewerage system including: the generation of shedders, the assignment of shedders to shared toilets that are connected to the hypothetical sewerage network, the amount of *S.* Typhi shed that enters and exits the sewerage system.

**Fig. A.1:**
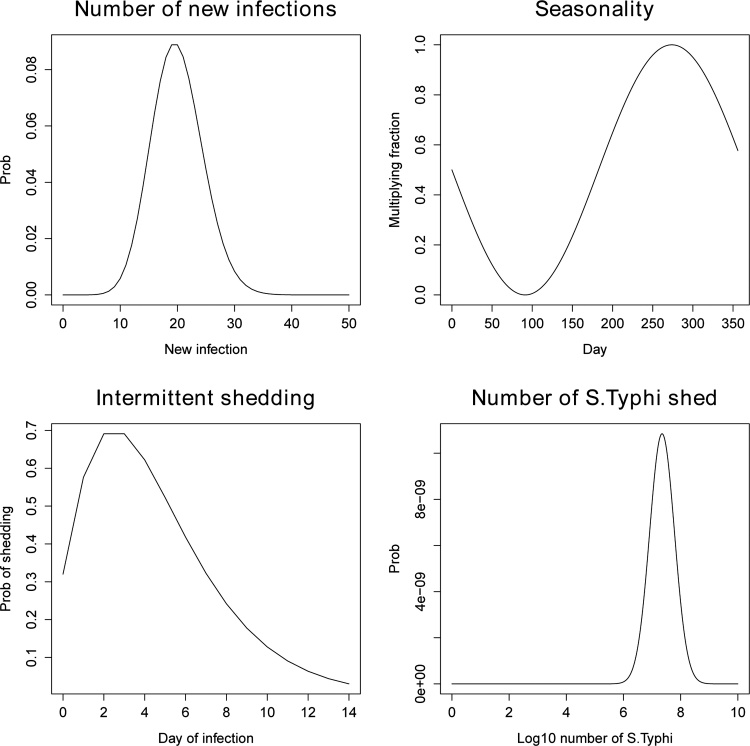
Settings and distributions for key factors including numbers of new infections per day (with λ=20), seasonality of typhoid fever infection incidence, intermittent shedding during the course of infection, and number of *S.* Typhi shed per day. The multiplying fraction, which depends on the day of the year, is a factor to multiply with the infection pressure.

The number of fecal pathogens entering the sewerage system varies by entry point (shared toilet) and day. *S.* Typhi was used as the model pathogen for the current simulation study. Fig. 2 shows how we simulated the shedding kinetics of *S.* Typhi. Distributions of key parameters are shown in Fig. A.1. The number of new infections (shedders) generated per day, X, was assumed to follow a Poisson distribution with a mean rate λ. Each new shedder was assigned to one of the 3000 shared toilets, and this shedder was assumed to continue shedding at the same location for the whole shedding period. Fig. 2 illustrates how the shedders were distributed in the network. First, real numbers from 0 to 1 were randomly divided into 3000 intervals ($\phi_{i}$) with unequal lengths to represent varying catchment sizes of 3000 toilets. The longer the interval, the better the accessibility and more people used this toilet. Second, the spatially explicit risks of infection, as weights, were multiplied by the length of intervals of those 3000 toilets to simulate geographic clustering of infection risk and *S.* Typhi shedding. Thus, each new shedder could be randomly assigned to a toilet with a probability proportional to the length of interval $\phi_{i}$. For each infection, shedding was assumed to last up to 14 days (Gibani et al., 2019). Seasonality could be simulated by multiplying λ with a periodic factor $-\frac{\sin(2\pi t/365)+1}{2}$, where t represents day of the year. In Kolkata, the monsoon season is the peak typhoid season (Sur et al., 2007, Saad et al., 2018). Intermittent shedding of *S.* Typhi (Young et al., 2015, Gibani et al., 2019) was simulated by assigning different probabilities of shedding to different days of infection. When an infected person is shedding, the number of *S.* Typhi bacteria shed, Y, was assumed to have a lognormal distribution with parameters $\left(\mu ,\sigma^{2}\right)$. In the simulation study, shedding from symptomatic and asymptomatic infections were not differentiated, and carrier status was not considered.

### 2.4 Pathogen flows, sampling, and detection

Pathogens entering the sewerage network are carried downstream towards the pumping station (Fig. 2). Residence time was assumed to be shorter than one day: any sewage entering the system was assumed to have completely left the network within 24 h. The fraction of the target pathogen lost through decay and leakage during the passage ranged from 0 to 1, depending on the time in the environment or travel distance in the sewerage, usually with a convex shape, which makes decay and leak faster at the beginning (Mahbub et al., 2004, Williams et al., 2005, Taylor et al., 2013). In this simulation study, pathogen loss was assumed to follow a gamma distribution with parameters (α,β) dependent on the distance traveled. With this decay and loss function, the number of pathogens passing any point in the sewerage network within a day can be determined. Since the volume of wastewater at any point in the sewerage network is known, the concentration of pathogens (daily average) at any point can be calculated.

Potential sampling sites were the 3000 shared toilets and the pumping station. Fecal pathogens at any point where a toilet connects to the sewer arrive from all upstream toilets (including itself). All pathogens pass through the pumping station, except those lost through decay or leakage. The simulation study examined three types of samples: (1) 40 L of sewage from the pumping station outlet; (2) 0.5 L sample of sewage from a single shared toilet; (3) 0.5 L pooled sample of sewage from five adjacent shared toilets (0.1 L each). The adjacent five shared toilets, where we collect the pooled sewage sample, were defined as a “primary sampling unit” (PSU). By averaging over multiple samples, any single shedder tends to have a higher probability of contributing to a pooled sewage sample. The lower limit of detection (LLOD) of the *S.* Typhi was set to $10^{3}$ genome equivalent copies (GEC) per sample based on pilot lab testing for *S.* Typhi in seeded sewage samples (unpublished data). During the pilot study, 40 L of sewage was collected at the pumping station and then concentrated by ultrafiltration so as to allow detection of concentrations higher than 25 GEC/L. For samples at shared toilets (single shared toilet or PSU), 0.5 L sewage samples were collected, allowing detection of concentrations higher than 2000 GEC/L.

### 2.5 Adaptive sampling site allocation

For ES, each sampling site represents a monitoring station that collects “signals” (positive or negative detection of target pathogen) with a certain frequency. Through adaptive sampling site allocation, the locations of sampling sites are dynamically updated to increase the probability of detecting a positive signal for the ES system. For the given model system, the adaptive algorithm can be deployed following the steps below.

**Initialization** The initial PSU sampling sites, denoted as $S_{0}=\{x_{1},x_{2},\ldots ,x_{n}\}$, can be selected using simple random sampling or stratified sampling. For simple random sampling, a rectangular area can be defined at a random GPS location, and five shared toilets (a PSU) can be randomly selected from the toilets within the rectangle. For stratified sampling, the geographic area is divided into a certain number of subareas and one PSU is randomly selected from each subarea. The advantage of stratified sampling is spreading out the sampling sites and maximizing the site coverage of the geographic area.

**Evaluation** After initialization, a certain number m of samples are collected at each sampling site (both pumping station and PSUs) resulting in a sequence of signals (positive and negative over time). The output from sampling site i at sample time j is denoted as $Z_{x_{i},t_{j}}$. Currently, we are interested in whether ES can be used to provide information on whether or not *S.* Typhi is circulating in the environment. If any of the sampling sites at time point j gets a positive signal, the output of the ES system ($Z_{t_{j}}$) is positive at that time point. For optimization, an information function I(S) can be defined as the information volume: the number of positive signals among m samples, when sampling a set of sites S.

A jackknife approach can be applied by removing one PSU at a time and evaluating the information loss: the number of positive signals lost if site $x_{i}$ is removed. This is a method to evaluate how important any individual site is to the entire ES system.

If $x_{i}$ is removed, the loss function can be defined as:

$$L(S,x_{i})=I(S)-I(S^{x_{i}})=\sum_{j=1}^{m}Z_{t_{j}}-\sum_{j=1}^{m}Z_{t_{j}}^{x_{i}}+\alpha \sum_{j=1}^{m}Z_{x_{i},t_{j}}+\epsilon ,$$

where $Z_{t_{j}}^{x_{i}}$ represents the output of ES system after removing site $x_{i}$ at time j. The primary information loss was considered as the loss in number of positives from ES system, $\sum_{j=1}^{m}Z_{t_{j}}-\sum_{j=1}^{m}Z^{x_{i}}$. The secondary information loss was considered as the number of positives lost from any individual site that is dropped, $\sum_{j=1}^{m}Z_{x_{i},t_{j}}$, and α represents the weighting coefficient to give smaller weight for secondary information loss. ϵ, which follows Unif(0,0.001), is the amount of noise to break any ties of information loss for different sampling sites.

**Update** When the information loss for removing any of the sampling sites is calculated, the PSU(s) with the smallest information loss will be dropped. The pumping station site, as a sampling site connected to the whole network, is a fixed sampling site that will never be dropped. After dropping any PSU site(s), new site(s) can be selected randomly, or based on additional information (e.g. location of clinical cases, upstream or downstream relationship), and added to the ES system for the next round of sampling. The combination of removing and adding is defined as an update of ES sampling sites ($S_{k}\to S_{k+1}$). Evaluation and update occur after every round of signal collection.

### 2.6 Simulation study

Two types of geographic distribution of risk were simulated: presence or absence of geographic clustering of risk. We added spatial variation in the risk of generating new infections (shedders) in the case of geographical clustering. Those two types of geographic distribution of risk, in combination with low or high pathogen loss, and low or high sensitivity of the assays to detect *S.* Typhi in the environmental samples, result in a total of eight scenarios. The infection pressure (λ) ranged from 1 to 200 average new shedders per day in all the scenarios.

For all eight scenarios, five sampling strategies were compared: pumping station (PS) only, 9 shared toilets, 9 PSUs, PS + 9 shared toilets, and PS + 9 PSUs, where 9 shared toilets or PSUs were located using the stratified sampling method described previously in the initialization phase above. Each simulation was for five years of ES, and 1000 iterations were run to generate a distribution of ES sensitivity (for each infection pressure) to evaluate the performance of the different sampling strategies. The ES sensitivity is the probability of detecting the *S.* Typhi released into the sewerage system by ES. For each iteration, we simulated shedding kinetics and pathogen flow for 2000 days. Adaptive sampling site allocation was applied for each of the eight scenarios with different infection pressures.

The different settings for the adaptive sampling site allocation method were compared to see how quickly the ES system would achieve high sensitivity. Five comparisons were examined:

1. Stratified sampling vs. simple random sampling in the initialization phase. Compared to simple random sampling, stratified sampling forces wider geographic distribution of the sampling sites.
2. Double the sample size in the initialization phase vs. normal sample size. Because it may take several rounds of update to increase the sensitivity of ES system to a high level, an efficient way to initialize the ES could be to start with a larger sample size for initialization (e.g. double the number of sampling sites) and then gradually decrease the sample size to normal sample size during the update phase.
3. Relocate two sites per update vs. relocate one site per update. The number of sites updated after each sampling round will influence the speed of adapting and the stabilization of ES sensitivity.
4. Collect 12 samples per update vs. 8 samples per update. With smaller number of samples collected per update, the speed of adapting will be faster. However, this setting has a higher risk of randomly dropping valuable sampling sites.
5. Collect weekly samples vs. collect samples every three days. With shorter time between each round of sample collection, the update process is faster. But this setting requires more lab capacity, including personal and equipment, to be able process the same number of samples within a shorter time period, and the autocorrelation in shedding may influence the results.

Optimal settings will need to consider the traderoffs between higher sensitivity vs. practical constraints (e.g. human resources, lab capacity, and cost).

Detailed parameters and settings of these simulations are listed in Table 1. All the simulations were run in R version 3.4.4 (R Core Team, 2015) on a computing cluster using Amazon Web Services (https://aws.amazon.com), and all the codes were published on Github (Source code on Github, 2019).

**Table 1:** Variables, distributions, parameters, and settings used in the simulation study of different scenarios.

	Variables	Distributions &parameters	References
*Ward 58 &59, Kolkata*			
· Population	$N_{\mathrm{pop}}$	160,000	Kolkata Municipal Corporation Website (2018)
· Number of households	$N_{\mathrm{hh}}$	30,000	Unpublished Data
· Number of shared toilets	$N_{\mathrm{tol}}$	3000	Estimated
· Water use per capita	$V_{\mathrm{use}}$	200 L/day	Basu (2015)
· Water loss	$Q_{l}$	35%	Basu (2015)
*Simulated sewerage network*			
· Number of pumping station	$N_{\mathrm{pump}}$	1	
· Number of edges	$N_{\mathrm{edge}}$	1000	
· Number of nodes (toilets)	$N_{\mathrm{node}}$	3000	
*Shedding dynamics*			
· Infection pressure	λ		
· Time (day)	t		
· Seasonality multiplier	$m_{s}$	$m_{s}=-\frac{\sin(2\pi t/365)+1}{2}$	
· Number of new infections	$X_{t}$	$\mathrm{Poisson}(m_{s}\lambda )$ generated on day t	
· Number of new infections generated on day t and entering at toilet i	$X_{t,i}$, i=1,2,…,n		
· Number of days infections duration	$T_{\mathrm{shed}}$	14 days	Gibani et al. (2019)
· Probability of shedding given the day of infection ($t_{\inf}$)	$P(\mathrm{shedding}|t_{\inf})$	NB(r=3,p=0.4)	Gibani et al. (2019), Assumption
		$P(\mathrm{shedding}|t_{\inf})=5\cdot \left(\begin{array}{c} r+t_{\inf}+1\\ t_{\inf} \end{array}\right)p_{\inf}^{t}{\left(1-p\right)}^{r}$	
· Number of *S.* Typhi shed (N) for the day given shedding	P(N|shedding)	Lognormal(μ=17.92,σ=1)	Expert Communication, Assumption
		$P(N=x|\mathrm{shedding})=\frac{1}{\sigma x\sqrt{2\pi }}e^{-{\left(\log(x)-\mu \right)}^{2}/2\sigma^{2}}$	
· Number of *S.* Typhi entering toilet i on day t	$N_{t,i}$, i=1,2,…,n		
· Percent of decay and loss of *S.* Typhi	$P(Q_{d}|d)$	$P(Q_{d}=x|d)=\frac{1}{\Gamma (k)\theta^{k}}x^{k-1}e^{-x/\theta }$	Assumption
$\left(Q_{d}\right)$ given the distance d		slow: k=1,θ=0.25	
		rapid: k=1,θ=2	
*Sampling*			
· Volume of samples	$V_{s}$	PS: 40 L	Pilot Study
		shared toilet: 0.5 L	
		PSU: 0.5 L	
· Number of toilets pooled per PSU	$n_{\mathrm{pool}}$	5	
· Number of sampling sites	$n_{s}$	PS: 1	
		shared toilet: 9	
		PSU: 9	
· LLOD of lab detection	$C_{\det}$	***low sensitivity***	Pilot Study
		PS: 25 GEC/L	
		shared toilet or PSU: 2000 GEC/L	
		***high sensitivity***	
		PS: 0.25 GEC/L	
		shared toilet or PSU: 20 GEC/L	
*Adaptive sampling site allocation*			
· Number of days per sample	$n_{\mathrm{days}/\mathrm{sample}}$	7	
· Number of samples per update	$n_{\mathrm{samp}/\mathrm{update}}$	12	
· Number of sites relocated per update	$n_{\mathrm{reloc}/\mathrm{update}}$	2	

## 3 Results

### 3.1 Comparison of ES sensitivity by sampling strategy

**Fig. 3:**
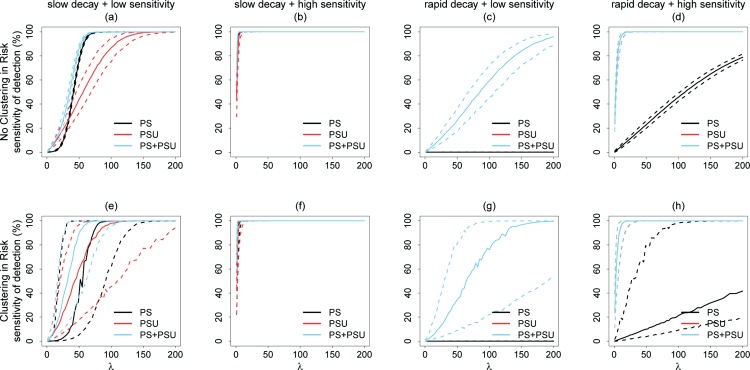
Sensitivities of different sampling strategies (PS, PSU, PS+PSU) under eight scenarios as a function of the infection pressure. The ES sensitivity is the probability of detecting the *S.* Typhi released into the sewerage system by ES. In the simulation, PS = collecting 1 sample at the pumping station for each time of sample collection. PSU = collecting 9 pooled toilet samples for each time of sample collection and each pooled sample is from 5 adjacent shared toilets. PS+PSU = collecting 1 pumping station sample and 9 pooled toilet samples for each time of sample collection. Eight scenarios are combinations of clustering of risk (yes/no), decay and loss rate in the environment (slow/rapid), and the sensitivity of the detection assay (low/high). The solid curve represents the median sensitivity of the ES system, while the dashed curves represent the 5th percentile and 95th percentile of sensitivity as calculated from 1000 iterations. λ is the infection pressure, which represents the expected (average) number of new shedders per day.

**Fig. 4:**
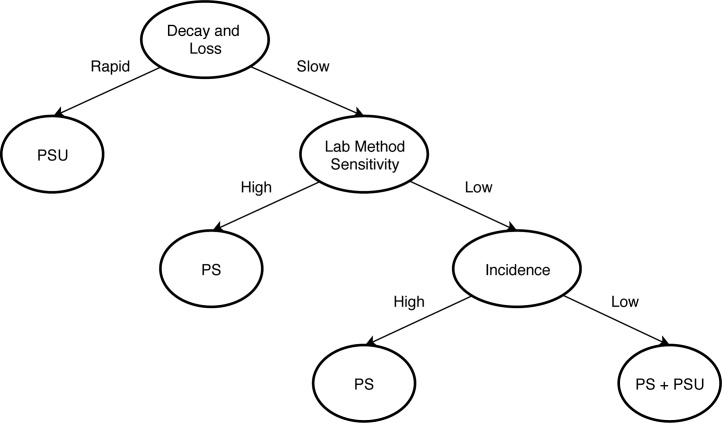
Decision tree for designing an environmental surveillance sampling strategy to optimize the target pathogen detection. PS = collecting samples at the pumping station. PSU = collecting pooled toilet samples from adjacent multiple shared toilets. PS+PSU = collecting both pumping station sample and pooled toilet samples. The choice of the appropriate sampling strategy depends on the pathogen loss in the environment, the sensitivity of the detection assay to detect the target pathogen in sewage, and incidence, which is a proxy for infection pressure.

**Fig. A.2:**
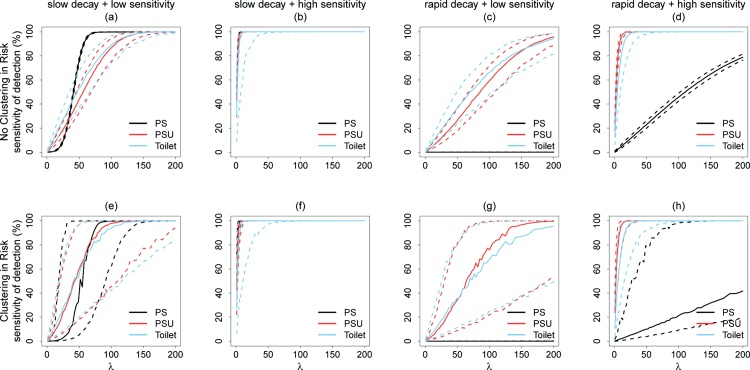
Sensitivities of different sampling strategies (PS, PSU, Toilet) under eight scenarios as a function of the infection pressure. The ES sensitivity is the probability of detecting the *S.* Typhi released into the sewerage system by ES. In the simulation, PS = collecting 1 sample at the pumping station for each time of sample collection. PSU = collecting 9 pooled toilet samples for each time of sample collection and each pooled sample is from 5 adjacent shared toilets. Toilet = collecting samples from 9 shared toilets for each time of sample collection. Eight scenarios are combinations of clustering of risk (yes/no), pathogen decay and loss rate in the environment (slow/rapid), and the sensitivity of the detection assay (low/high). The solid curve represents the median sensitivity of the ES system, while the dashed curves represent the 5th percentile and 95th percentile of sensitivity as calculated from 1000 iterations. λ is the infection pressure, which represents the expected (average) number of new shedders per day.

Fig. 3(a)–(d) show the sensitivity of *S.* Typhi ES for three sampling strategies (PS, PSUs, PS+PSUs) for four scenarios without geographic clustering of risk. Infection pressure, λ, represents the expected average number of new shedders per day. When pathogen loss was low (slow decay, little leakage) and the sensitivity of the detection assay was low (Fig. 3(a)), sampling strategies based on PSU sites tended to have higher ES sensitivity compared to the PS site under low infection pressure. As the infection pressure increased and there were more shedders in the community, samples from the PS site provided higher sensitivity. When pathogen loss was low and the sensitivity of the detection assay was high (Fig. 3(b)), the PS site and the PSU sites performed equally well. When pathogen loss was high (Fig. 3(c) and (d)), the PSU sites performed better than the PS site regardless of the sensitivity of the detection assay. The PS site barely contributed any additional information, which made the performance of the PSUs strategy vs. the performance of the PS+PSUs strategy basically the same. When pathogen loss was high and the sensitivity of the detection assay was low, the sensitivity of PS site remained very low level even when the infection pressure increased because the model predicted that most of the *S.* Typhi would die off or leak out of the sewers before arriving at the pumping station. Fig. 3(e)–(h) show the sensitivity of those three sampling strategies for the same four scenarios with geographic clustering of risk. The patterns were similar to those when no spatial clustering was present except the variation in ES sensitivity was greater for all the sampling strategies and scenarios. Meanwhile, the sensitivity of the ES based on PS site tended to increase more slowly, while the sensitivity of the ES based on PSU sites increased faster as the infection pressure increased. The results can be summarized as a decision tree for designing a sampling strategy (Fig. 4). In addition, the results comparing PS, PSUs, and single shared toilets sampling strategies are shown in Fig. A.2.

### 3.2 Improvement from adaptive sampling site allocation on ES sensitivity

**Fig. 5:**
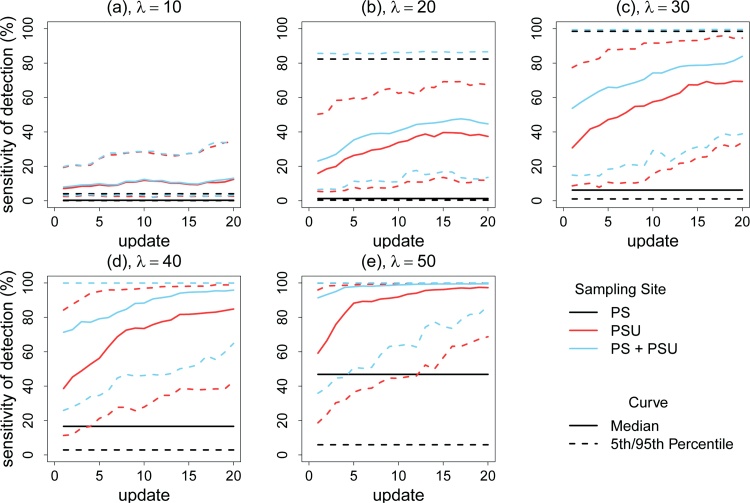
Predicted ES sensitivity of detecting the *S.* Typhi by number of updates, given slow pathogen decay and loss, low sensitivity of the detection assay, and geographic clustering of risk under 5 different infection pressures. In the simulation, PS = collecting 1 sample at the pumping station for each time of sample collection. PSUs = collecting 9 pooled toilet samples for each time of sample collection and each pooled samples from 5 adjacent shared toilets. PS+PSUs = collecting 1 pumping station sample and 9 pooled toilet samples for each time of sample collection. The solid curve represents the median sensitivity of the ES system, while the dashed curves represent the 5th percentile and 95th percentile of sensitivity as calculated from 1000 iterations. λ is the infection pressure, which represents the expected (average) number of new shedders per day.

**Fig. A.3:**
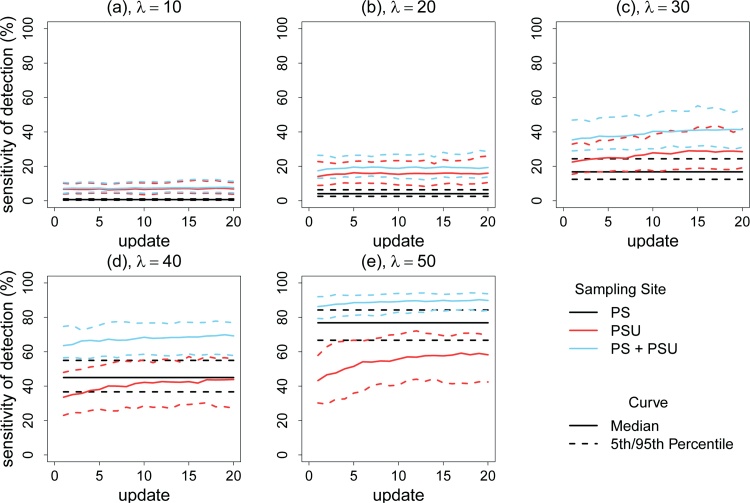
Predicted ES sensitivity of detecting the *S.* Typhi by number of updates, given slow decay and loss, low sensitivity of detection assay, but no geographic clustering of risk under 5 different infection pressures. In the simulation, PS = collecting 1 sample at the pumping station for each time of sample collection. PSU = collecting 9 pooled toilet samples for each time of sample collection and each pooled samples from 5 adjacent shared toilets. PS+PSU = collecting 1 pumping station sample and 9 pooled toilet samples for each time of sample collection. The solid curve represents the median sensitivity of the ES system, while the dashed curves represent the 5th percentile and 95th percentile of sensitivity as calculated from 1000 iterations. λ is the infection pressure, which represents the expected (average) number of new shedders per day.

Fig. 5 shows the ES sensitivity curves over the course of 20 updates with low pathogen loss, low sensitivity of the detection assay, and geographic clustering of risk, while Fig. A.3 shows the equivalent curves without geographic clustering of risk. The λ (infection pressure) was set at 10, 20, 30, 40, or 50 new shedders per day. Without geographic clustering of risk (Fig. A.3), the adaptive sample allocation improved the sensitivity when λ was high (≥30). However, the sensitivity of ES based on the PS site was higher than the sensitivity of the ES based only on PSU sites when λ was high. When there was geographic clustering of risk (Fig. 5), there were substantial boosts in sensitivity using adaptive sample allocation for all λs. For example, when λ was set at 30 new shedders/day (Fig. 5(c)), the median ES sensitivity increased from 53.7% to 83.9% in 20 updates for the PS + PSUs sampling strategy. The sensitivity of ES when based on PSU sampling sites was always better than that for ES based one the PS site.

### 3.3 Sampling site heatmap

**Fig. 6:**
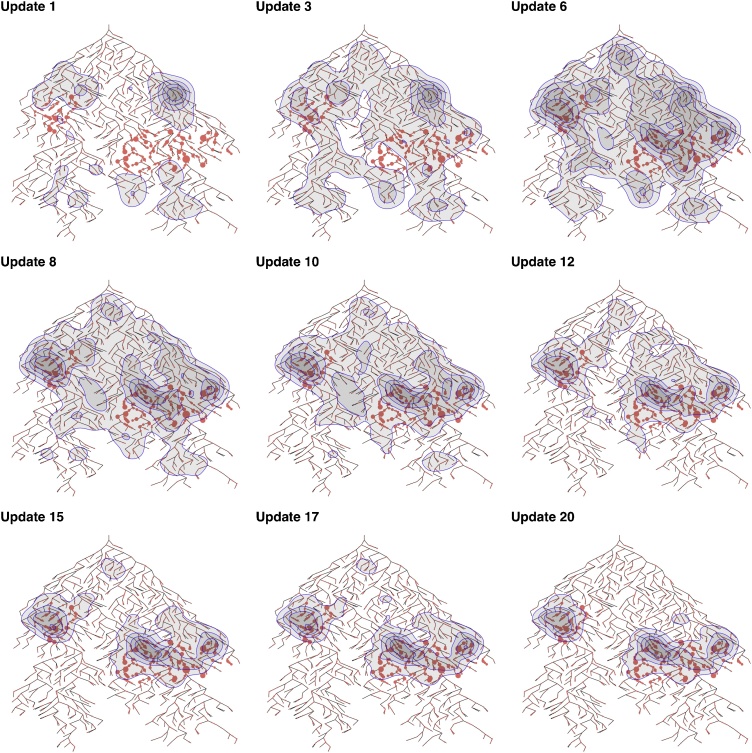
Heatmaps of sampling site distributions along with the update process given λ=40 new shedders per day. The heatmap has two layers: the base layer has the hypothetical sewerage network and toilets. The red dots represent the shared toilets, and the size of a red dot represents the probability of a *S.* Typhi shedder defecating at this toilet. The second layer shows the contours of sampling sites distribution in blue. The contours of sampling sites will change with update process and over time will move to the geographic locations with the most shedders. (For interpretation of the references to color in this figure legend, the reader is referred to the web version of this article.)

An alternative way to visualize the performance of the adaptive sampling site allocation method is by using heatmaps. Fig. 6 shows dynamic heatmaps of sampling site distribution (contours) over the locations of *S.* Typhi shedders in a hypothetical sewerage network. This shows how the adaptive sampling site allocation method leads to prioritizing sample collection in geographic areas with the highest numbers of shedders. When the samples from the PS site are negative for *S.* Typhi, the best PSU or shared toilet sampling sites are located at high-risk areas which are close to the shedders. Despite limited observations (positive/negative results from a limited number of sampling sites), the sampling sites recommended by the model converged to the high-risk areas for most scenarios, except for very low infection pressure (λ≤10).

### 3.4 Optimal settings for adaptive sampling site allocation

For the five settings compared in Table 2 and Fig. 4, Fig. 5, Fig. 6, we found that doubling the sample size in the initialization phase and relocation of two sites per update allowed the update process to reach high sensitivity faster. Stratified sampling in the initialization phase provided slightly higher sensitivity at the start of the update process. Eight samples collected per update performed equally well compared to 12 samples collected per update when the infection pressure was at least 30 new shedders per day. When the infection pressure was low, 12 samples per update performed slightly better than 8 samples per update. Collecting samples every three days performed equally well as weekly samples.

**Table 2:** Comparing the performance of adaptive sampling site allocation with different settings under two infection pressures (λ=20 new shedders per day; λ=40 new shedders per day). The ES sensitivity is the probability of detecting the *S.* Typhi released into the sewerage system by ES. The reference group used the following settings: stratified sampling with normal sample size (9 PSU sites) initialization, relocate two sites per update, 12 samples collected each site per update, and collect samples every week. The simulation was conducted separately for each pair of settings (e.g. Collect sample every week vs. three days).

			Sensitivity at initialization in % median (95% credible interval)	Sensitivity after 10 updates in % median (95% credible interval)	Sensitivity after 20 updates in % median (95% credible interval)
λ**= 20**	**Initialization**	Reference	**18.1 (5.6, 47.4)**	32.8 (7.2, 64.6)	40.1 (11.0, 73.1)
		Simple Random Sampling	15.5 (4.6, 43.3)	30.3 (7.9, 61.9)	40.6 (10.5, 70.3)
		Reference	16.5 (5.3, 46.0)	32.4 (7.6, 62.2)	40.5 (10.3, 71.7)
		Double Sample Size	**36.2 (17.1, 67.9)**	**39.4 (11.1, 73.0)**	**43.8 (14.0, 76.9)**
	**Update**	Reference	16.9 (5.2, 44.6)	**31.8 (7.4, 62.0)**	**39.3 (11.3, 73.3)**
		Relocate one site per update	16.3 (5.2, 43.4)	25.2 (6.6, 55.4)	31.4 (7.4, 62.1)
		Reference	16.3 (5.5, 44.9)	**31.2 (7.4, 62.5)**	**40.0 (11.2, 71.6)**
		8 samples collected per update	16.8 (5.2, 47.9)	28.8 (6.8, 63.6)	35.8 (8.6, 71.9)
		Reference	15.5 (5.1, 47.9)	31.9 (7.6, 65.4)	40.6 (11.4, 73.8)
		Collect samples every three days	16.5 (5.0, 46.7)	30.2 (6.7, 63.2)	39.8 (11.0, 73.8)

λ**= 40**	**Initialization**	Reference	**42.7 (12.1, 88.3)**	77.8 (28.2, 97.9)	89.2 (53.1, 99.3)
		Simple Random Sampling	40.5 (11.1, 86.7)	78.6 (28.8, 98.0)	90.6 (53.8, 99.3)
		Reference	45.7 (13.0, 88.6)	79.0 (29.2, 98.0)	90.2 (57.1, 99.3)
		Double Sample Size	**77.9 (40.1, 97.8)**	**88.5 (50.5, 99.3)**	93.5 (66.7, 99.7)
	**Update**	Reference	44.4 (13.4, 89.6)	**78.4 (30.3, 98.2)**	**90.4 (53.2, 99.4)**
		Relocate one site per update	44.7 (12.7, 89.7)	68.2 (21.3, 96.5)	80.9 (34.5, 98.3)
		Reference	43.2 (12.2, 89.0)	78.1 (32.6, 97.8)	90.4 (58.2, 99.2)
		8 samples collected per update	46.3 (12.8, 89.3)	79.5 (30.6, 97.3)	88.8 (51.4, 99.2)
		Reference	42.6 (12.5, 85.4)	78.2 (33.5, 97.4)	89.4 (54.8, 99.3)
		Collect samples every three days	44.9 (12.5, 87.9)	78.4 (31.5, 97.8)	90.1 (57.2, 99.2)

## 4 Discussion

Environmental surveillance has proved to be a valuable tool in the polio eradication campaign and is now being considered for other disease control programs where clinical surveillance is challenging. To link the occurrence of enteric infections in the population with presence of enteric pathogens shed in the environment, various kinds of information are needed, including the numbers of infected subjects, the number of pathogens shed by any infected subject, and the environmental fate of these pathogens. In ES, the sensitivity of the detection assay used for pathogen detection, the number of sampling sites, and the frequency of sampling are also limiting factors. Therefore, the choice of where to sample is critical for the success of an ES program (Kalkowska et al., 2019). A simulation model can be helpful by predicting the probability of detecting any shedders in the population under different scenarios given a specific sampling strategy.

We present a simulation model of pathogen fate in an urban environment, where excreted pathogens enter a sewerage network that serves the entire population and the pathogens, travel from small peripheral branches towards wider conduits to finally pass through a pumping station. This model was demonstrated to be instrumental in understanding the relation between pathogens detected in sewage and the infected subjects who excreted these pathogens. As sewer lines merge into main branches, the size of the connected population increases, but both the travel time through the sewerage system and the dilution of the pathogens in the sewage increase. Therefore, the relation between sampling locations and ES sensitivity of detection is not straightforward.

All the pathogens that enter the sewerage anywhere in the system will pass through the pumping station (if they are not lost or inactivated during their journey). However, when collecting and analyzing samples solely from the pumping station, the sensitivity of the ES system is only high under certain favorable conditions: slow pathogen decay, highly sensitive detection method, and high incidence of infection in the population. Sampling at peripheral locations, especially pooled samples from shared toilets (PSU), yield better sensitivity under certain scenarios, and when shedders are clustered (spatially, temporally) the chance of detection increases. The choice of where to sample is critical, and our results show that an optimizing strategy may be used to increase the probability of finding positive samples.

### 4.1 Sampling strategies

Environmental surveillance has several unique characteristics that must be considered when designing a sampling strategy. First, the ES sampling sites function as monitoring stations, and samples are routinely collected from the same locations. Second, pathogen sources vary in location, time, and magnitude. Third, the sewerage network structure generates correlations in the collected data (e.g. between upstream samples and downstream samples). Finally, pathogen-specific characteristics such as decay rate in the sewage environment and the sensitivity of the detection assay, will influence the ability of the ES system to detect infections.

In this simulation study, we explored eight scenarios with different geographic distributions of shedders, pathogen loss (due to decay and leakage) in the environment, and sensitivity of detection assays under various infection pressures. The results are summarized in a decision tree for designing an ES sampling strategy (Fig. 4):

1. When there are substantial losses out of the sewerage network or pathogen decay, only a small proportion of the shed pathogens may reach the pumping station where the sewage will also be highly diluted. In such cases, collecting sewage samples close to the locations of shedders will be more sensitive, even though the catchment population is smaller.
2. When pathogen loss is high and the detection method is highly sensitive, sampling at the pumping station should still be sufficient to detect the target pathogen despite travel time and dilution. However, this scenario approaches an ideal situation which may rarely occur in practice.
3. When pathogen loss is low, but the detection assay has a low sensitivity, the choice of sampling strategy should be based on infection pressure. When the infection pressure is expected to be high, there are more infections in the population and more pathogens entering the environment at multiple points in the network. In this situation, sampling at the pumping station is still appropriate. When the infection pressure is expected to be low, sampling at the pumping station is less sensitive than sampling at PSUs due to the higher dilution at central locations like the pumping stations. Therefore, sampling at both PSUs and the pumping station is the best strategy.

In Kolkata, a city with moderately high typhoid incidence (Ochiai et al., 2008), we chose to use a PS+PSUs sampling strategy for a pilot ES due to lack of information about pathogen decay, leakage in the sewage lines, and geographic distribution of typhoid infections in the city. After several rounds of sampling, the sampling strategy, the number of sampling sites, and the locations of sampling sites will be adjusted based on the results of samples collected.

Disease surveillance is a dynamic process: whenever any of the critical factors in the decision tree changes (Fig. 4), the ES sampling strategy should be refined or adjusted. This is extremely important, as interventions such as vaccination or improved sanitation, may decrease infection pressure. If the ES is not strategically designed, a decreased incidence of shedders may result in failure to detect low levels of circulating pathogens. Low sensitivity of the ES system also makes it more difficult to certify elimination or eradication of a disease.

### 4.2 Adaptive sampling site allocation

When a sampling strategy has been chosen there are still ways to improve the sensitivity of ES. In case the sensitivity of the detection assay is low, and the infection pressure is low, the sensitivity of ES can be increased by adding more sampling sites or increasing the sampling frequency. However, there options will require additional human resources and lab capacity.

Adaptive sampling site allocation was developed to improve the sensitivity of ES given a fixed number of sampling sites. The adaptive sampling approach has been used to predict disease prevalence (Kabaghe et al., 2017) and conduct mosquito surveillance (Sedda et al., 2019) for malaria. For polio ES, the quality of any sampling site is validated on whether any enterovirus are detected at that site or if poliovirus is detected in at least 50% of samples within a 6-month period (The Global Polio Eradication Initiative, 2018). Sites that do not meet these criteria are dropped or replaced. (Kroiss et al., 2018) There are two critical reasons to adapt the sampling site locations. First, infection pressure and areas of risk may not be well characterized during the initial ES design phase. An adaptative approach transforms ES into a dynamic system and uses the detection information from the previous round of samples to adjust the sampling strategy for the next data collection stage. Second, disease dynamics and shedding kinetics vary in time and space, and consequently the infection pressure may change and high-risk areas may relocate.

When the infection pressure is very low (λ≤10 new shedders per day) it is still possible to improve ES sensitivity by using adaptive sampling site allocation, but the updates are more or less random due to negligible differences in information loss for multiple sites. For λ between 10 and 50 new shedders per day, there can be a strong improvement in the sensitivity of ES within the first 20 updates from the initial sites. The best sampling sites for PSUs or single shared toilets are in high-risk areas, or a short distance downstream from high-risk areas. By applying the adaptive selection process, the sampling sites converge towards high-risk areas after several rounds of sampling and updating (Fig. 6).

As the total number of samples is often constrained by logistics and resources, the main challenge of the allocation method is how quickly it achieves sufficiently high sensitivity given a fixed sample size. The simulation study tested various strategies. In order to define a good initial set of sampling sites, two methods were examined. Stratified sampling for initialization provides better spatial coverage while tending to quickly capture high-risk areas. Use of a double sample size initialization provides an additional boost in sensitivity at the start of ES. During the update process after double sample size initialization, the adaptive sampling site allocation method maintains sensitivity while the sample size decreases by selecting the sites that contribute the most information. By relocating additional sites at each update step, and collecting samples more frequently, the increase in sensitivity can be accelerated. However, an increased number of samples will also increase the workload and costs of the ES program.

Fig. 5 shows that at high infection pressures, the sensitivity increased more rapidly during the update process. It should be noted that the infection pressure is likely to show seasonality. Therefore, having more updates and more frequent sample collection during a time with the high infection pressure will result in more rapid improvements in ES sensitivity and locating high-risk areas. A practical scenario for the deployment of ES could be: (1) initialize with stratified sampling and double sample size during the high incidence disease season (i.e. when infection pressure is high); (2) relocate more than one site per update and increase sampling frequency during the high incidence season; (3) save resources by conducting less frequent sampling and reduce update frequency during the low incidence season.

### 4.3 Clinical surveillance vs. environmental surveillance

Enteric infections can be transmitted directly (person-to-person) or indirectly (contaminated environment, drinking water, or food). Pathogens shed by infected individuals can be ingested by susceptible subjects via multiple pathways, creating a transmission cycle. Enteric disease surveillance may target different parts of this cycle.

Clinical surveillance is based on the detection of cases with symptoms serious enough to be recognized, diagnosed, and reported. Currently, active community-based clinical surveillance for typhoid is conducted in some parts of Kolkata, India. The sensitivity of the active community-based surveillance may be quite good. Some may argue that this is the gold standard, but it is labor-intensive and expensive. The sensitivity of hospital based surveillance, especially passive surveillance, is likely to be low due to underreporting, under-ascertainment, because of non-specific symptoms, lack of a sensitive diagnostic test for typhoid infection, and unreported medical intervention (e.g. self-treatment with antibiotics) that can affect diagnosis. Therefore, clinical surveillance of illnesses with low incidence and/or non-specific symptoms tends to underestimate the incidence. A fundamental characteristic of clinical surveillance is that asymptomatic infections and carriers of typhoid fever cannot be detected. Because asymptomatic shedders and carriers also contribute to the infection pressure, knowledge about their prevalence is valuable for public health.

ES is based on the detection of the target pathogen shed by infected people into the environment, by examining their presence in catchment reservoirs of community feces. In urban areas, feces are usually collected in the sewerage infrastructure or in shared on-site sanitation (pit latrines or septic tanks). In rural areas, there may be shared on-site sanitation and open defecation. Monitoring the presence of the target pathogen in sewage therefore informs about the circulation of the target pathogen in the population served by the sewerage system. Monitoring pathogens in a compartment more proximal to ingestion, like drinking water, can be useful, to estimate the potential risk associated with a specific transmission pathway. However, this approach does not provide an estimate of infection burden in the community because drinking water is not directly from the compartment where shed pathogens enter the environment.

Enteric disease surveillance may require different strategies depending on context. When the enteric disease is endemic with high disease incidence, clinical surveillance can provide accurate disease information including spatial and temporal patterns and inform vaccine campaigns. When the incidence of symptomatic cases is reduced, for instance as a result of a successful intervention (e.g. improved water and sanitation or vaccination), it may be too low to effectively monitor by clinical surveillance. When the incidence of infection is low, exposure decreases and the fraction of symptomatic infections also tends to decrease (Teunis et al., 2018). Due to asymptomatic infections, there may still be pathogens shed into the environment and ES may be more critical for detecting a “silent” burden of infection. When moving towards disease elimination, the sample size of ES should be increased to achieve greater sensitivity to detect low numbers of the target pathogen circulating in sewage and more power to verify elimination. The method described in this paper is useful to guide sampling strategies and sampling site allocation at different stages of ES.

ES is increasingly recognized as a low-cost, sustainable alternative or possible supplement to clinical surveillance of enteric pathogens. Comparing clinical and environmental surveillance in multiple contexts for different pathogens will be critical to validate the value of ES (Cowger et al., 2017).

Given this adaptive sampling approach to optimize sampling site allocation and ES sensitivity, is ES for *S.* Typhi likely to be sensitive enough to help inform *S.* Typhi control programs? Environmental surveillance has been critical to the poliomyelitis eradication campaign (Hovi et al., 2012), and it is logical to consider applying this strategy to inform control measures for other enteric pathogens. Both poliovirus and *S.* Typhi are excreted exclusively by infected humans and have no environmental reservoirs. During acute infection, the amount of target pathogen shed per gram of stool is similar for both poliovirus and *S.* Typhi (ranging from $10^{2.57}$ to $10^{7}$ cell culture infectious dose for poliovirus and mean $10^{5}$ viable *S.* Typhi cells) (Buonagurio et al., 1999, Wain et al., 2008), and the duration of shedding overlaps for both pathogens (up to 8 weeks for wildtype poliovirus vs. up to 3–4 weeks for *S.* Typhi) (Buonagurio et al., 1999, Vollaard et al., 2005). However, there are a number of fundamental differences between the *S.* Typhi bacterium and *S.* Typhi infection and poliovirus and poliovirus infection that are likely to affect the sensitivity and value of ES for *S.* Typhi. Decades of vaccine campaigns have dramatically reduced infection with wildtype poliovirus, so it is likely that any population will have far fewer (if any) people shedding wildtype poliovirus compared to *S.* Typhi. Persistence of enteric pathogens in sewage is affected by many environmental factors (temperature, sunlight, pH, association with particles, etc.), and there is very limited information on both poliovirus and *S.* Typhi survival in sewage. Poliovirus is fairly stable in wastewater and has been reported to remain detectable for 3 months in sewage (Dowdle and Birmingham, 1997), and Enriquez-Enriquez (1994) reported that the time for a 99% reduction of poliovirus in primary effluent at 15 °C was 28 days. Limited data on *S.* Typhi persistence indicate that the bacteria can be detected in pond water for up to 12 days and groundwater for up to 20 days (Cho and Kim, 1999). *S.* Typhi survival in sewage is likely to be shorter because of indigenous microbial activity. Finally, detection of poliovirus in sewage samples is quite sensitive, and the WHO ES method has been reported to detect 10–20 50% Tissue culture Infective Dose (TCID50) of poliovirus in a 500 ml grab sample using cell culture amplification (World Health Organization, 2003). In contrast, culturing *S.* Typhi from environmental samples has proved to be challenging, possibly because the bacteria enter a viable but non-culturable state (Cho and Kim, 1999). Our lab has recently reported detection of *S.* Typhi DNA in sewage by realtime quantitative PCR (Kapoor et al., 2019) with a range of detection limits. The sensitivity (limit of detection) and specificity of methods to detect *S.* Typhi in sewage will be a critical determinant of the success of a sewage surveillance system to provide useful information on typhoid prevalence in a city.

### 4.4 Strengths and limitations

This simulation study demonstrated a novel approach for the design, and adaptive improvement, of an ES system for *S.* Typhi in an urban setting.

First, we show how it is possible to explore different sampling strategies to optimize ES sensitivity before investing in sample collection and analysis. Five different sampling strategies (PS, single shared toilets, PSUs, PS+single shared toilets, PS+PSUs) were simulated, predicting the performance for eight scenarios with different geographic distribution of risk, pathogen loss (decay and leakage) in the environment, and sensitivity of detection assays. Each scenario can be tested with a range of infection pressures (λ) from 1–200 new shedders/day. Second, the mathematical model used for this simulation has a set of parameters that can be easily modified to conduct similar studies for other locations and other diseases. The simulation model could also be potentially applied to explore the effect of more robust ES (in cases averted) given a specific intervention like a vaccination campaign or improved sanitation. Furthermore, the comprehensive numeric results were simplified into a decision tree to better guide the selection of sampling sites for ES. Finally, the adaptive sampling site allocation method was demonstrated to provide improved sensitivity, dynamically adapting spatial and temporal changes in shedder prevalence and disease transmission.

Although the model allows adjustment of the infection pressure temporally and spatially to consider seasonality and relocation of high-risk areas, the influence of these variations on sampling strategies and selection of sampling locations was not explored in the current study. Also, the influence of the structure of sewerage network on the results was not examined. Likewise, the shedding dynamics of subjects with symptomatic and asymptomatic infections were not differentiated, and carrier status of typhoid was not considered. Variations within a day for some factors, including the number of circulating pathogens and the volume of sewage passing through any point in the sewerage network, were also not considered. Finally, the effect of rainfall on sewage dilution and flow velocity was not included.

Information gaps on *S.* Typhi and the sewerage network were explored using simulation with different scenarios. Addressing information gaps (e.g. persistence of *S.* Typhi DNA in the sewage and limit of detection in sewage) will lead to better decisions about ES sampling strategy and more accurate predictions of infection burden in the population.

## 5 Conclusion

This paper describes the application of a simulation study to explore different scenarios for ES and test a proposed sampling site allocation method. The results from the simulation study show that the optimal ES sampling strategy for a specific fecal pathogen can be determined systematically using information such as disease incidence, pathogen loss (decay and leakage) in the environment, and sensitivity of detection assays. A novel adaptive sampling site allocation method was developed to optimize sampling site locations and boost the sensitivity of ES despite constraints on sample size and frequency of sampling. The model and proposed method is general and can be used to guide sewage ES for any enteric pathogen and support decision-making about investment in disease control programs.

## Declaration of interests

The authors have all certified there is no potential conflict of interest.

## Figures and Tables

**Fig. A.4:**
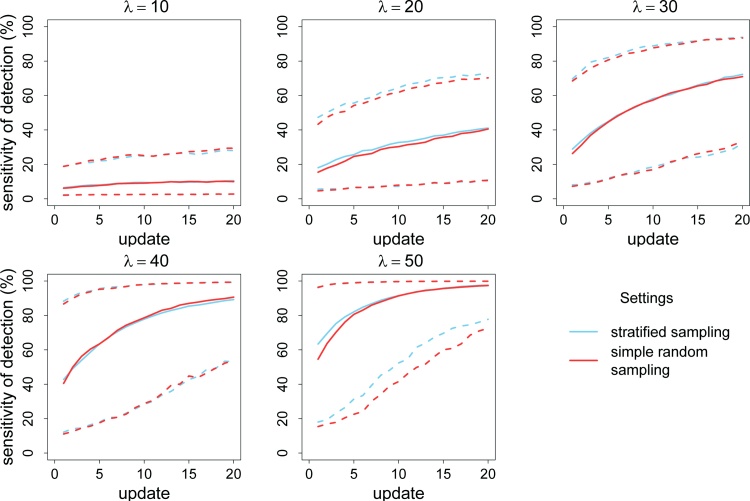
Comparison of predicted ES sensitivity of detecting the *S.* Typhi by number of updates between stratified sampling vs. simple random sampling in the initialization phase. The solid curve represents the median sensitivity of the ES system, while the dashed curves represent the 5th percentile and 95th percentile of sensitivity as calculated from 1000 iterations. λ is the infection pressure, which represents the expected (average) number of new shedders per day.

**Fig. A.5:**
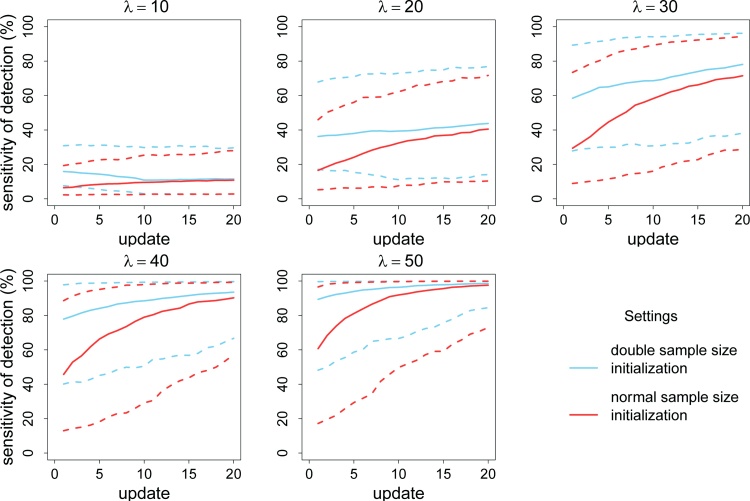
Comparison of predicted ES sensitivity of detecting the *S.* Typhi by number of updates between double sample size vs. normal sample size in the initialization phase. The ES sensitivity is the probability of detecting the *S.* Typhi released into the sewerage system by ES. The solid curve represents the median sensitivity of the ES system, while the dashed curves represent the 5th percentile and 95th percentile of sensitivity as calculated from 1000 iterations. λ is the infection pressure, which represents the expected (average) number of new shedders per day.

**Fig. A.6:**
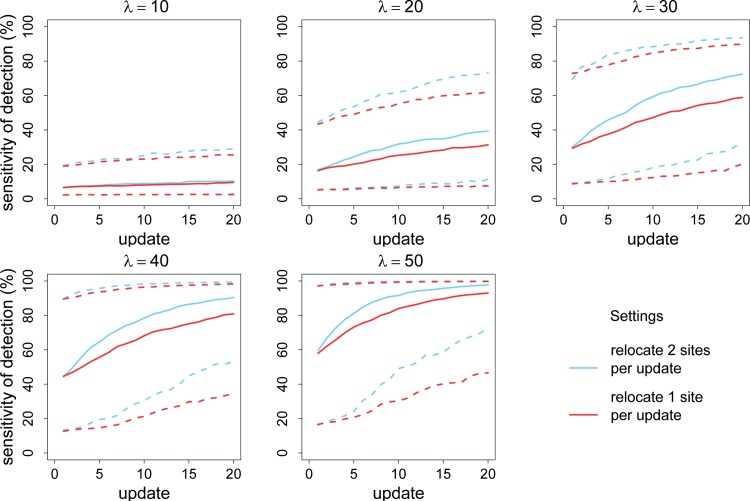
Comparison of predicted ES sensitivity of detecting the *S.* Typhi by number of updates between relocating two site vs. relocating two sites per update. The solid curve represents the median sensitivity of the ES system, while the dashed curves represent the 5th percentile and 95th percentile of sensitivity as calculated from 1000 iterations. λ is the infection pressure, which represents the expected (average) number of new shedders per day.

**Fig. A.7:**
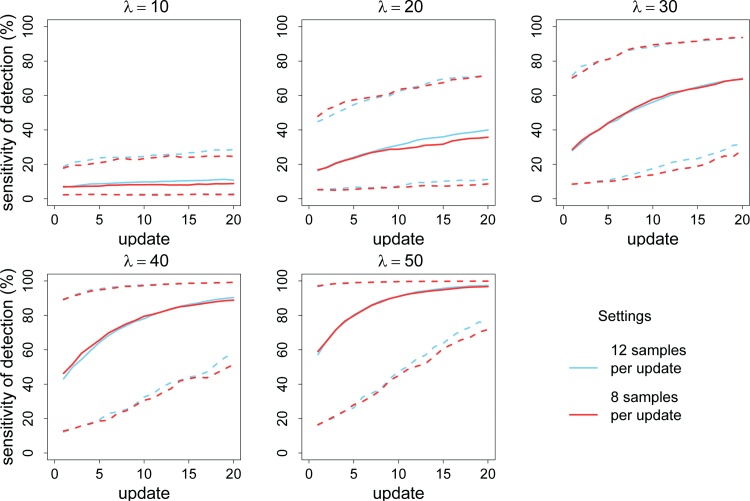
Comparison of predicted ES sensitivity of detecting the *S.* Typhi by number of updates between collecting 8 samples per update vs. collecting 12 samples per update. The solid curve represents the median sensitivity of the ES system, while the dashed curves represent the 5th percentile and 95th percentile of sensitivity as calculated from 1000 iterations. λ is the infection pressure, which represents the expected (average) number of new shedders per day.

**Fig. A.8:**
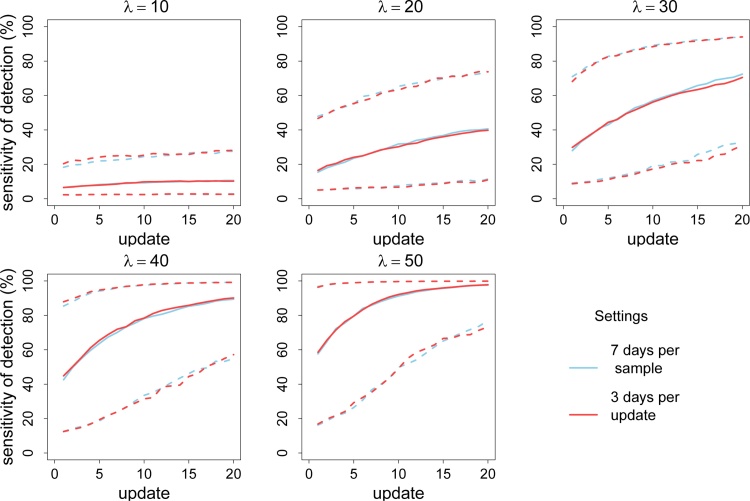
Comparison of predicted ES sensitivity of detecting the *S.* Typhi by number of updates between collecting weekly samples vs. collecting samples every three days. The solid curve represents the median sensitivity of the ES system, while the dashed curves represent the 5th percentile and 95th percentile of sensitivity as calculated from 1000 iterations. λ is the infection pressure, which represents the expected (average) number of new shedders per day.

## Appendix A

### A.1 Additional graphs

Fig. A.1 Settings and distributions for key factors
Fig. A.2 Sensitivity of detection for PS, PSU, and Toilet sampling strategies
Fig. A.3 Sensitivity curves along with the update process given slow decay and loss, low lab method sensitivity, but no geographic clustering of risk
Fig. A.4 Comparison of stratified sampling vs. simple random sampling in initialization phase
Fig. A.5 Comparison of double sample size vs. normal sample size in initialization phase
Fig. A.6 Comparison of relocating one site vs. two sites per update
Fig. A.7 Comparison of collecting 8 samples per update vs. collecting 12 samples per update
Fig. A.8 Comparison of collecting weekly samples vs. collecting samples every three days
